# Shortening delays in seeking aid for cardiovascular events: a step beyond

**DOI:** 10.1016/j.lansea.2024.100369

**Published:** 2024-02-27

**Authors:** Xi Lang, Zining Zhu, Yinqi Qian, Vicente Artola Arita, Tieying Zeng

**Affiliations:** a Deptartment of Nursing, Tongji Hospital Affiliated to Tongji Medical College, Huazhong University of Science and Technology, Hubei Wuhan, 430030, China; b Nursing School, Tongji Medical College, Huazhong University of Science and Technology, Hubei Wuhan, 430030, China; c Department of Global Public Health and Bioethics, Julius Center, University Medical Center (UMC) Utrecht, Utrecht, the Netherlands

Worldwide, cardiovascular diseases (CVD) are the leading cause of morbidity and mortality, and remain the top two causes of years of life lost after the age of 50.^1^^,^^2^ Ischemic heart disease and ischemic stroke were attributed more than 12 million deaths, according to data across 204 countries and territories by 2021.^2^ In the last three decades, CVD incidence has increased by over 100% in middle- and low-income countries despite a global decline in age-standardized incident rates.^3^ This increase may be explained by a low percentage of controlled traditional risk factors, namely hypertension and non-high-density lipoprotein cholesterol.^4^^,^^5^ Moreover, regional factors that delay a timely referral or intervention may increase CVD-related deaths.

To delve into regional factors of delay in seeking CVD intervention, Krishnan et al. conducted a community-based study investigating delays in seeking appropriate care for people dying from CVD events in a community in northern India and explored the causes and determinants of such delays.^6^ The “three delay” model was adopted to investigate delays in seeking/receiving care, and a mixed-effect logistic regression analysis and qualitative interview were utilized. Krishnan and colleagues add information as to why the majority of patients die out-of-hospital due to level-1 and -2 delays and inspires future tailored interventions in India to shorten the time of delays in care (Fig. 1).

**Fig. 1:**
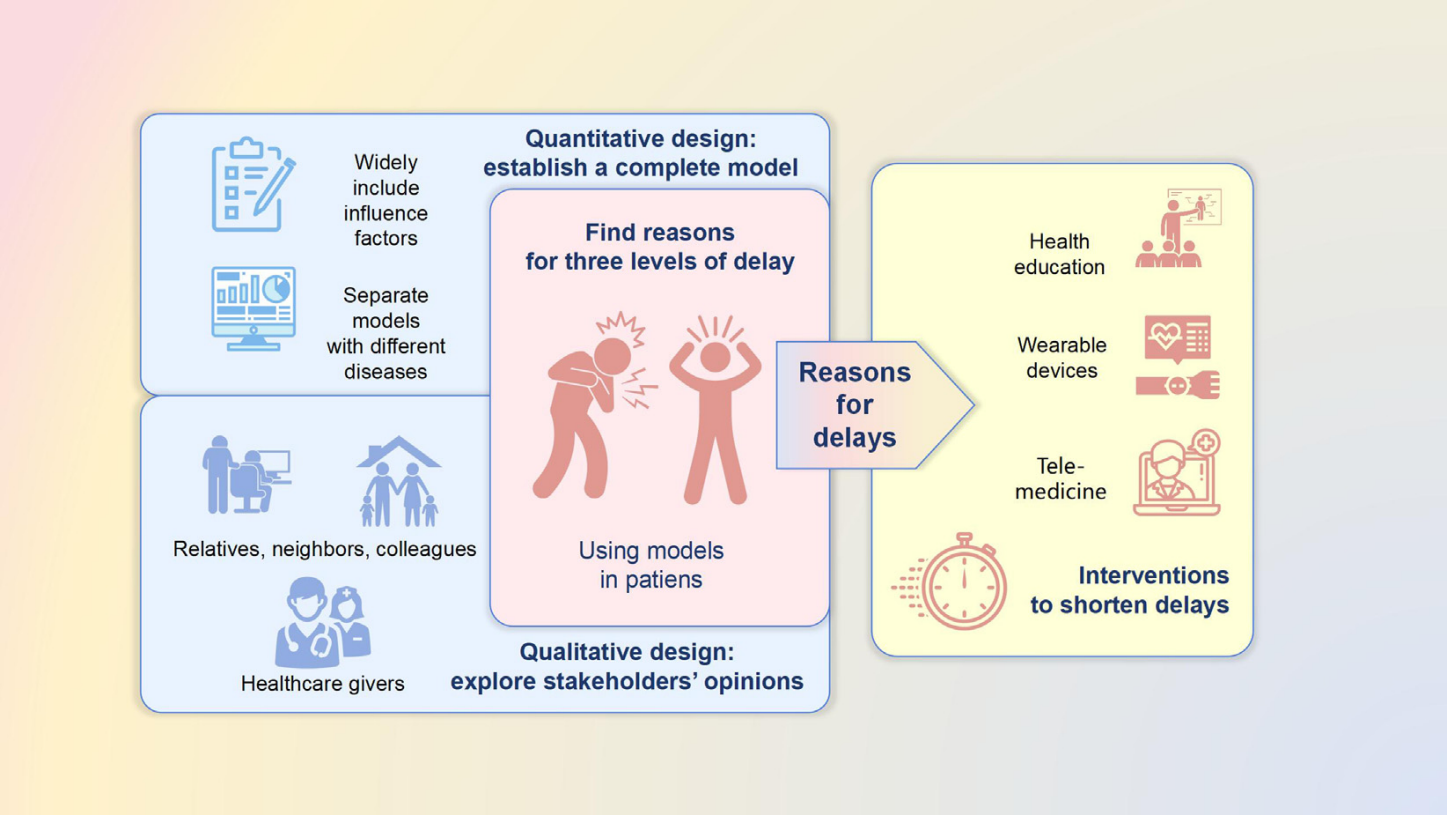
From research to practice: accurately reducing delays.

However, study design limitations are essential to address in both quantitative and qualitative approaches. First, the conditions of death cases are pretty multiple, such as essential hypertension, hypertensive heart disease, hypertensive heart and renal disease, ischemic heart disease, cardiomyopathy, other forms of heart disease, subarachnoid hemorrhage, and stroke. Although they all fall under the cardiovascular and cerebrovascular diseases category, the specific clinical manifestations vary depending on the disease types. The generalizability of conclusions is constrained by the varied impacts that influencing factors may exert on each disease.

Second, traditional risk factors associated with CVD events were not included in data collection, constraining the results of the mixed-effect logistic regression on their influence.^2^ Consequently, future studies should also incorporate traditional risk factors and adjust for them in the logistic analysis to mitigate confounding effects and yield more robust evidence regarding the studied association.^1^^,^^2^

Third, logistic regression combining stroke and cardiac event cases may have leant the results towards cardiac event cases due to the imbalance of fewer stroke cases. It raises the question of whether the results represent factors applicable to both conditions separately. Although the researchers explained that the number of cases with a single disease was insufficient for logistic regression, future studies may extend the time of data collection or incorporate multiple centers. The results of a single outcome logistic regression will be more targeted to facilitate precise interventions and shorten care delays.

Fourth, a higher likelihood of delays in stroke cases was asserted, particularly at levels 2 and 3. Nevertheless, distinctions in delays between stroke and cardiac emergencies were substantiated through the Chi-square test, a method designed to address distribution disparities. To provide more comprehensive insights into the factors influencing delays in seeking/receiving care, further conclusive findings will be derived through a logistic regression analysis.

Finally, the complementary and detailed qualitative approach by Krishnan and colleagues helped integrate the possible causes of delay and the judgment of the actual delay. However, based on the limited number of specialists interviewed, all plausible delay causes may have yet to be explored. Future qualitative research initiatives may explore the causes of level-1 and-2 delays broadly by interviewing more junior professionals, such as community primary care providers and ambulance first responders.^7^

A step beyond in research may use the “three delay” model to identify interventions to shorten delays in seeking aid for cardiovascular events at each level. Such interventions may include raising awareness of alarming symptoms through health education, monitoring of at risk-patients with wearable devices, and providing remote medical care to diagnose suspicious patients.^8^, ^9^, ^10^ (Fig. 1).

## Contributors

XL, ZNZ, and YQQ have conceptualized, written and edited the comment. VAA and TYZ reviewed the draft and final manuscript.

## Declaration of interests

The authors declare no conflicts of interest regarding this article's research, authorship, or publication.
